# Why are people with high self-control happier? The effect of trait self-control on happiness as mediated by regulatory focus

**DOI:** 10.3389/fpsyg.2014.00722

**Published:** 2014-07-08

**Authors:** Tracy T. L. Cheung, Marleen Gillebaart, Floor Kroese, Denise De Ridder

**Affiliations:** Self-Regulation Lab, Department of Clinical and Health Psychology, Utrecht UniversityUtrecht, Netherlands

**Keywords:** trait self-control, regulatory focus, promotion, prevention, happiness

## Abstract

**Background:** While self-control has often been related to positive outcomes in life such as higher academic achievements and better health, recent insights reveal that people with high trait self-control (TSC) may even experience greater life satisfaction or happiness.

**Objective:** The current study further scrutinizes this potential association between TSC and happiness, and examines how regulatory focus, defined as the way people frame and direct their goal pursuit strategies, plays a role in this relationship. Accordingly, the present study examines the mediating role of regulatory-focus (promotion and prevention focus) on the relationship between TSC and happiness.

**Method:** Data was collected from 545 individuals (65.9% female, *M*_age_ = 27.52 years) regarding their TSC, regulatory focus, and happiness.

**Results:** Mediation analyses demonstrate that TSC positively predicts happiness, while this effect was partially mediated by relatively more promotion focus and less prevention focus.

**Conclusion:** Results suggest that people with higher TSC are happier possibly because they are: (1) more promotion-focused on acquiring positive gains thereby facilitating more approach-oriented behaviors, and (2) less prevention-focused on avoiding losses thereby reducing avoidance-oriented behaviors. These findings are relevant for topical scientific debates regarding the underlying mechanisms of self-control regarding initiatory and inhibitory behaviors.

## INTRODUCTION

Self-control is defined as the capacity to alter and regulate predominant response tendencies resulting in the inhibition of undesirable behaviors while promoting desirable ones to support the pursuit of long-term goals (de Ridder et al., 2012). On a dispositional level, trait self-control (TSC) is a basic temperament forming the core of personality as it develops (Rothbart et al., 2000), and research has also consistently shown higher TSC to be associated with more positive outcomes in life such as higher academic achievement, better health, more interpersonal success, and less maladaptive adjustments (Tangney et al., 2004). As such, self-control has been heralded as an evolutionary trait to ensure adaptation and survival (Baumeister et al., 2007). Nonetheless, while substantial research has shown the long-term beneficial effects of TSC on various domains of life, much less is known about its relation to happiness. As a central tenet of self-control involves the trade-off between relishing immediate gratifications and achieving long-term goals, it is interesting to know where happiness stands in this transaction – how does TSC affect happiness and why? The present research examines the relationship of TSC and happiness and introduces different ways of framing goal pursuit (regulatory focus, Higgins, 1997) as a potential mediator in this relationship. With this research we aim to contribute to the currently limited understanding of the relationship between TSC, goal pursuit framing, and happiness.

Although it is apparent that people with higher TSC are more successful in life, are they also happier? One could imagine that constantly self-regulating according to morals, standards, and social expectations would result in living a dull, mundane, and joyless life. Lending support to this speculation, emotion regulation research has shown that indeed individuals with high TSC experience less momentary affect, inhibited expression of spontaneity and extraversion (Zabelina et al., 2007), as well as limited emotional intensity on a day-to-day basis (Layton and Muraven, 2014). Accordingly, these findings would speculate that having a high disposition of TSC might hold people back from experiencing happiness to the fullest in life.

However, Hofmann et al. (2013) have recently ruled out this speculation by revealing that individuals with higher TSC are not only happier in that they experience greater life satisfaction, they also do not need to self-regulate as often as one may think. Indeed, while having to forego an immediate pleasure for a distant, long-term goal may not be a pleasant emotional experience, findings indicate that people high in TSC generally experience problematic desires on fewer instances, and are therefore less prone to the emotional distress engendered by such self-control trade-offs. Furthermore, the authors have shown that although people with high TSC are not immune to motivational conflicts, they are much more successful in managing competing goals by favoring the one with the more virtuous outcome. Together, these results suggest that having a high disposition of TSC enables individuals to experience less problematic desires, and manage goal pursuit better in favor of the more virtuous goal, which in turn results in more happiness in life. Nonetheless as this study remains to be the first and only study to date to provide direct evidence that TSC positively influences happiness, more research is warranted.

Accordingly, the current study aims to extend the research regarding the relationship between TSC and happiness by questioning how TSC is related to goal framing. Given that TSC is a basic temperament forming the basis of personality (Rothbart et al., 2000) and that it is the initial capacity that enables individuals in long-term goal pursuit, there is reason to speculate that TSC correspondingly affects the way people *frame* their goals and goal pursuit strategies. In this line of reasoning, the present study explores regulatory focus, which is the orientation of goals in terms of a promotion or prevention focus, in relation to TSC and happiness.

### REGULATORY FOCUS AND SELF-CONTROL

According to Higgins’ (1997) regulatory focus theory, people have two motivational orientations that direct their goal pursuit behaviors: promotion and prevention. A promotion focus is concerned with growth, advancement, and accomplishment. Accordingly, goals are framed as gains and non-gains, and *approach* goal pursuit strategies that strive toward positive outcomes are favored. Moreover, promotion focus encourages the reduction of *errors of omission* (i.e., missing out on an opportunity to accomplish something; Crowe and Higgins, 1997). Meanwhile, a prevention focus is preoccupation with vigilance, responsibilities, and oughts. Goals are therefore framed in respect to losses and non-losses, and *avoidance* goal pursuit strategies that deter from *errors of commission* (i.e., making mistakes) are preferred.

Regulatory focus also plays an important role in the manner that people experience and resolve a motivational conflict (Scholer and Higgins, 2010). For example, a dieter’s goal to lose weight could be represented as an ideal or aspiration in a greater promotion-focus, whereas it could be considered as a duty or obligation in a greater prevention-focus. When exposed to a tempting chocolate cake, greater promotion-focus may lead a dieter to endorse approach strategies to advance to the goal – “I want to be physically fit” or “My goal is to eat healthily,” whereas greater prevention-focus may encourage strategies to resist the temptation by avoiding it – “I ought to stay away from this chocolate cake” or “I should not eat sweets.” As such, successful goal pursuit for a greater promotion focused individual may be about following one’s dreams and aspirations, whereas for a greater prevention focused individual this may be about living up to one’s duties and obligations with minimal mistakes.

Recent research by Lisjak and Lee (2014) suggests that regulatory focus may be influenced by one’s self-control levels. The authors have found that when individuals were induced to a state of low self-control, they exhibited enhanced motivation for protection. In explaining such finding, the authors posited that the experience of low self-control heightened perceived vulnerability, and consequently increased individuals’ motivation for protection and vigilance characterized by a prevention-focus orientation. In a similar vein, we also speculate TSC to be associated with promotion and prevention focus, respectively, in which individuals correspond the orientation of their regulatory focus to be compatible with their TSC levels.

### CURRENT STUDY

In the present study we propose that TSC initiates regulatory focus that frames the actual goal pursuit strategies (i.e., promotion focus with approach-oriented strategies or prevention focus with avoidance-oriented strategies), which then relates to happiness. Considering that TSC is a basic temperament that forms the foundation of personality (Rothbart et al., 2000) and the effects of experimentally manipulated self-control on regulatory focus (Lisjak and Lee, 2014), we conceptualize TSC as an antecedent to regulatory focus. Correspondingly, our view is that TSC is the initial capacity for long-term goal pursuit, and regulatory focus is the corresponding strategy for actualizing the goal pursuit. As such, our reasoning aligns with Lyubomirsky et al.’s (2005) model of happiness, which proposes that people can enhance their happiness through intentional strategies, such as pursuing personally concordant goals, but only under the assumption that they have the prerequisite self-control capacity to first initiate these strategies.

Accordingly, we hypothesize that TSC positively relates to happiness with its effect mediated (at least partially) by regulatory focus. Although no previous research has specifically explored the mediating role of regulatory focus on the effect of TSC on happiness, some speculations are made about the nature of these relationships. As promotion focus is associated with aspirations, it could be argued that having more TSC that favors long-term goals would initiate approach-oriented behaviors toward the goal; hence, a positive relation between TSC and promotion focus could be assumed. Subsequently, greater promotion focus should increase the likelihood of actualizing the goals of aspirations and ideals, and the result of achieving these positive gains would increase happiness. Therefore, it is predicted that higher TSC is positively related to promotion focus, and in turn promotion focus positively predicts happiness.

As for prevention focus, however, the predictions are less obvious as, conceptually, both positive and negative associations with TSC may be plausible. On the one hand, as prevention focus endorses a mindset of vigilance and is considered typical for TSC, a positive association between TSC, and prevention focus could be assumed. On the other hand, the recent findings by Hofmann et al. (2013) have suggested that people high in TSC are less occupied with staying away from temptations, thereby implying a negative relation between TSC and prevention focus. Furthermore, prevention focus could be assumed to be a negative predictor of happiness because avoidance-oriented behavior away from vices may cause one to forego many momentary pleasures for the sake of achieving higher order long-term goals. However, it is also equally arguable that prevention focus is assumed to be a positive predictor of happiness because one could ultimately take joy and satisfaction from the valued long-goal achievement despite having to give up on the short-term gratifications during the goal pursuit.

In summary, we expect the effect of TSC on happiness to be mediated by regulatory focus, but while we expect TSC to be positively associated with promotion focus, we do not draw any specific hypothesis for the direction in which prevention focus mediates the relationship between TSC and happiness. By exploring the mediating role of regulatory focus on the effect of TSC on happiness, the present study aims to contribute to the current limited literature on TSC and happiness, as well as to add to the understanding of the underlying mechanisms of self-control by exploring its relation to regulatory-focus.

## MATERIALS AND METHODS

### PARTICIPANTS AND PROCEDURE

The sample consisting of 546 participants were Internet users from Germany who participated in a larger study that assessed personality dimensions (see Denissen and Penke, 2008). The mean age of the participants was 27.52 (SD = 11.01), and females made up of 65.9% of the sample. Moreover, 4.5% of the sample did not receive any formal education, 50.2% were educated up to high-school level, and 45.2% were enrolled in or had completed a university degree. Participants completed measures on TSC, regulatory focus including both promotion focus and prevention focus, and also happiness. Accounting for missing data, the final sample consisted of 523 participants.

### MEASURES

#### Trait self-control

Finkenauer et al.’s (2005) short version of the Self-control Scale was administered to measure TSC. Participants indicated the degree to which they agreed (1 = *entirely false*, 5 = *entirely true*) with 11 statements such as “I am good at resisting temptations” and “I am lazy” (reverse coded). A final TSC score was calculated by averaging the scores from all items, where a higher score indicated higher TSC. A Cronbach’s alpha (α) of 0.80 reported good internal consistency for the 11-item short version of Self-control Scale in the current study.

#### Regulatory focus

Regulatory focus was assessed with the Regulatory Focus Questionnaire (Lockwood et al., 2002), which consisted of two subscales to measure both promotion and prevention focus. Participants indicated their response to 18-items on a 9-point scale with end points 1 (*not at all true of me*) and 9 (*very true of me*). Sample items include “I frequently imagine how I will achieve my hopes and aspirations” and “I frequently think about how I will prevent failures in my life.” Separate measures of promotion focus and prevention focus were created by averaging the scores belonging to each subscale, and a higher score reflected greater focus strength. Both subscales demonstrated good internal consistency (promotion focus: α = 0.78; prevention focus: α = 0.82) of the Regulatory Focus Questionnaire in the current study.

#### Happiness

The Subjective Happiness Scale (Lyubomirsky and Lepper, 1999) was employed where participants indicated the degree to which they agreed to each of the four statements. For example, on a five-point scale participants rated how “In general, I consider myself” (1 = *not a very happy person*; 5 = *a very happy person*), and “Some people are generally not very happy. Although they are not depressed, they never seem as happy as they might be. To what extent does this characterization describe you?” (1 = *not at all*; 5 = *very much*; reverse coded). A final happiness score was calculated by averaging all the scores from all individual items, where a higher score represented greater happiness. Cronbach’s alpha (α = 0.87) indicated good internal consistency for the Subjective Happiness Scale in the current study.

## RESULTS

### DESCRIPTIVES

Overall participants exhibited moderate levels of TSC (*M*= 2.96, SD = 0.65). On average, participants reported relatively greater promotion focus (*M*= 3.56, SD = 0.67) compared to prevention focus (*M*= 2.84, SD = 0.79), *t*(524) = 16.45, *p*< 0.001. Finally, participants also indicated relatively high levels of happiness (*M*= 4.47, SD = 1.49). All intercorrelations of the study variables are presented in **Table 1**.

**Table 1:** Intercorrelations between study variables.

	1	2	3	4	5
Age (1)	–	–	–	–	–
TSC (2)	0.22^**^	–	–	–	–
Promotion focus (3)	–0.05	0.21^**^	–	–	–
Prevention focus (4)	–0.24^**^	–0.48^**^	0.06	–	–
Happiness (5)	0.19^**^	0.50^**^	0.31^**^	–0.51^**^	–

** Correlation is significant at the 0.01 level (2-tailed).

### MAIN ANALYSES

**FIGURE 1:**
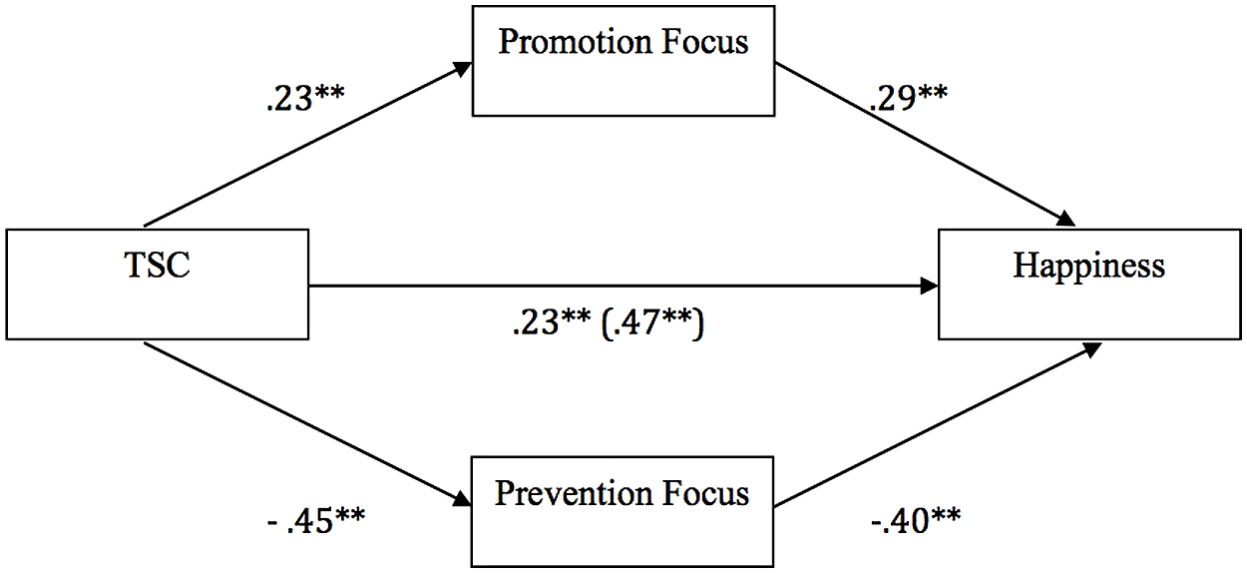
**Results of mediation analyses testing promotion focus and prevention focus as mediators of the effect of TSC on happiness while controlling for age as a covariate.** Double asterisks (**) indicate coefficients are significantly different from zero, *p* > 0.001. When the mediators are included, the coefficient changes from β = 0.47 to β = 0.23, indicating partial mediation.

A mediation model was built to test whether the effect of TSC on happiness was mediated by regulatory focus. A series of regression equations relating TSC (the independent variable), promotion focus and prevention focus (the potential mediators), and happiness (the dependent variable) were performed using bootstrapping analyses (based on 5,000 bootstrap samples) in the SPSS macro (PROCESS; model 4) recommended by Hayes (2013). Additionally, as age was significantly correlated with happiness [*r*(523) = 0.19, *p*< 0.001)], it was included as a covariate in the mediation analyses. The results of the analyses are depicted in **Figure 1**.

Results were in line with our prediction that TSC was positively associated with promotion focus [β = 0.23, 95% CI (0.14, 0.32)]. Moreover, results also revealed that TSC was negatively associated with prevention focus [β = -0.45, 95% CI (-0.52, -0.37)]. Furthermore, while greater promotion focus predicted more happiness [β = 0.29, 95% CI (0.22, 0.36)], less prevention focus predicted more happiness [β = -0.40, 95% CI (-0.47, -0.32)]. TSC had an indirect effect on happiness through promotion focus [β = 0.07, 95% CI (0.04, 0.10)] and prevention focus [β = 0.18, 95% CI (0.23, 0.23)] respectively. Accordingly, the combined indirect effect that TSC had on happiness through promotion and prevention focus as mediators was β = 0.24, 95% CI (0.19, 0.30). Finally, the direct effect of TSC on happiness remained significant [β = 0.23, 95% CI (0.15, 0.31)], therefore indicating partial mediation by promotion and prevention focus.

## DISCUSSION

In our daily lives, we encounter different obstacles in the form of temptations that promise immediate gratifications and momentary pleasures that may impede our long-term goal pursuit. Recent findings by Hofmann et al. (2013) have revealed that while people with high TSC encounter these motivational conflicts less, they are also better at managing competing goals by favoring the one with the more virtuous outcome, which in turn leads to greater life satisfaction. Extending these findings, our present study provides results suggesting that individuals with high TSC orient their goal pursuit strategies tactfully according to promotion and prevention regulatory focus, which is then related to happiness. Our results revealed two distinct patterns of mediation through a promotion focus and a prevention focus. On one hand, TSC positively relates to promotion focus, and greater promotion focus is associated with more happiness. On the other hand, TSC negatively relates to prevention focus, and less prevention focus is associated with more happiness.

Our findings complement previous research positing that individuals with high TSC experience problematic desires and temptations infrequently as they strategically structure their lives to steer clear away from these vices (de Ridder et al., 2012; Hofmann et al., 2012). If individuals with high TSC are less likely to encounter motivational conflicts, they are therefore also less obligated to exert avoidance-oriented strategies associated with a prevention focus to resist or counter temptations or vices. Instead, they are more liberated to pursue their goals, aspirations and ideals by carrying out approach-oriented strategies to actualize their personal ambitions as encouraged by a promotion focus. On the other hand, although it could be plausible that prevention oriented avoidance behaviors away from vices and giving up on hedonic pleasures during the goal pursuit may not be affectively pleasant, occasional execution of these avoidance behaviors are nonetheless essential to provide meaning and support for an individual in their goal pursuit of long-term goals. While it is known that success in goal pursuit brings positive affect (Oatley and Johnson-Laird, 1987), it is not difficult to imagine why people with high TSC are happier – they have greater dispositional capacity and more opportunities to facilitate goal achievement.

Furthermore, our findings also offer insight into the underlying mechanisms of TSC in resolving motivational conflicts. Traditionally self-control has been conceptualized as the capacity to withstand immediate gratifications and momentary pleasures, where emphasis was placed on the inhibition of response tendencies (e.g., Baumeister et al., 1998; Tangney et al., 2004). As such, self-control has been described as “an avoidance-oriented situation” (Muraven, 2008, p. 769). Interestingly, our results showing TSC to be negatively related to prevention focus and positively related to promotion focus suggests that TSC is not preoccupied with vigilance characterized by a prevention focus (as posited by the traditional conceptualization of TSC), but that TSC also highly involves eagerness that is facilitated by a promotion focus. In light of this, our findings lend support to the contemporary perspective that acknowledges both inhibitory and initiatory components of self-control (de Boer et al., 2011; de Ridder et al., 2011). According to this perspective, goal achievement can be accomplished in two ways. On one hand, TSC directs *inhibitory* behaviors to resist temptations or undesirable behaviors that hinder the long-term goal (e.g., restraining from excessive alcohol consumption). On the other, TSC invests toward *initiatory* behaviors that make advances to the long-term goal (e.g., initiating more physical exercise). Considering this, our finding that higher TSC is associated with greater promotion focus and relatively less prevention focus helps to characterize the discernment between initiatory and inhibitory self-control behaviors as described above. While more promotion oriented approach behaviors resembling initiatory self-control are associated with greater happiness, the reverse pattern is apparent for prevention-oriented behaviors that resemble inhibitory self-control. After all, refraining oneself from giving into desires is not the same as striving toward valued goals, as one may argue that happiness derives from actual goal achievements.

The study of TSC in relation to happiness is a relatively new topic, and our study is the first to our knowledge to examine the mediating role of regulatory-focus on the effect of TSC on happiness. By employing the Subjective Happiness Scale (Lyubomirsky and Lepper, 1999) to measure happiness directly, our study provides additional support for Hofmann et al. (2013) initial finding that TSC is related to life satisfaction. As such, our present research contributes to the current limited literature on the relationship between TSC and happiness by demonstrating the role of TSC and regulatory focus in relation to happiness. Moreover, as the substantial size of the sample captured a wide diversity in the population, there is confidence that our results are generalizable to the greater public.

Nonetheless, limitations to the study should be addressed. First, the collected responses were based on self-report measures, and hence there may be a possibility that social desirability could have biased the responses regarding self-control, regulatory focus and happiness (Van de Mortel, 2008). It could be possible that participants over report their levels of TSC and happiness as these are considered to be desirable constructs. Similarly, considering that promotion focus is associated with positive affectivity and that prevention focus is associated with negative affectivity (Summerville and Roese, 2008), this may have biased participants to ascribe higher levels of promotion focus and lower levels of prevention focus to themselves. As a result, such response bias could have potentially contributed to our pattern of findings and we address this as a limitation of the self-report measures used in the current study.

Second, as our data was cross-sectional and correlational in nature, this precludes drawing causal statements about the observed relationships between TSC, regulatory focus and happiness. Consequently, our data could not rule out the possibility that on the statistical level regulatory focus is the antecedent before TSC, rather than a mediator, in predicting happiness. However, on a theoretical level, TSC in the current study is conceptualized as the initial *capacity* to alter and regulate predominant response tendencies in order to achieve long-term goals, while regulatory focus is considered as the corresponding *strategy* to actualize the goal pursuit. We also consider TSC as antecedent before regulatory focus, as TSC has been argued to be a basic temperament that forms the foundation of developing personality (Rothbart et al., 2000). Moreover, we also take into account the recent study by Lisjak and Lee (2014) which has found participants to adopt a heightened sense of self-protection motivation subsequent to having their self-control levels to be reduced. The authors explained their findings by positing that having low self-control enhances perceived vulnerability, which in turn activates a prevention-focus orientation associated with vigilance. Relating back to our research, it could be possible that individuals have to adapt their regulatory focus or goal-orientation in order to match their TSC or their capacity for goal pursuit, and the association between the two thereby relates to happiness. While the findings of Lisjak and Lee’s (2014) study provide indirect evidence for our theoretical outlook, we nonetheless recommend future investigations to adopt a more rigorous experimental design to include both state and trait levels of self-control in a longitudinal design, in conjunction with regulatory-focus in affecting happiness.

We also welcome future studies to assess other constructs in conjunction with TSC in relation to happiness. For example, Conscientiousness as a Big Five personality trait (Costa and McCrae, 1992) has been demonstrated to predict subjective well-being and life-satisfaction (e.g., Keyes et al., 2002; Hayes and Joseph, 2003; Weiss et al., 2008). In explaining the importance of Conscientiousness for happiness, it has been suggested that individuals high on conscientiousness are more likely to behave effectively in achieving their goals (e.g., planning ahead, fulfilling commitments), and that goal achievement ultimately results in more subjective well-being (Hayes and Joseph, 2003). Given the close relationship between TSC and Conscientiousness (Tangney et al., 2004), and how both these constructs positively relate to goal pursuit, it would be interesting to explore how Conscientiousness predicts happiness in conjunction with TSC.

## CONCLUSION

Self-control has been linked to successes in different walks of life, and it appears that with greater self-control one could focus more on aspirations and less on warding off hindrances along the way. That said, although the pursuit of happiness may not be easy, it appears to be nonetheless in our control.

## Conflict of Interest Statement

The authors declare that the research was conducted in the absence of any commercial or financial relationships that could be construed as a potential conflict of interest.
